# The Tsetse Fly

**Published:** 1896-08

**Authors:** 


### EDITORIAL.

---

The office of the Business Manager of THE JOURNAL is Nos. 311 and 312 Fitten Building.

The editors are not responsible for views expressed by contributors.

Articles for publication should reach this office not later than the 15th inst.

Address all communications, and make all remittances payable to THE ATLANTA MEDICAL AND SURGICAL JOURNAL, Atlanta, Ga.

Reprints of articles will be furnished, when desired, at a nominal cost. Requests for the same should always be made *on the manuscript*.

---

### THE TSETSE FLY.

The African continent has suffered more than any other, even than Australia, from difficulties of transport which have kept it, though a close neighbor of Europe and Asia, a laggard in the race for civilization, and won for it the uncomplimentary title of the Dark Continent. Its rivers, owing to the step-like manner in which the high interior plateaux are reached, have afforded little help, for, generally speaking, they are blocked a few hundred miles from the sea by impassable waterfalls; and its low lying coast is frequently deadly to Europeans, while its inhabitants for the most part are warlike savages. But beyond these, it possesses in the tsetse fly perhaps a more powerful barrier to the entrance of the light of civilization than any of these others. It has been said in this connection, that "it is an ill wind that blows nobody good," and hypocritical and jealous editors and others, who profess to see in European intervention in Africa nothing better than "land piracy and territorial greed," apparently find pleasure in dwelling on the above obstacles to this advance of the white man, preferring that the heart of Africa should be eaten out by bloodthirsty Prembehs, Arab slave-dealers, and murdering Matabeles, and forgetting that if priority of occupation be the only criterion of the right to proprietorship, irrespective of form of government and the character of the people, these same self-righteous critics are among the thieves and robbers who have done the aborigine out of his natural birthright.

The tsetse fly (*Glossina morsitans*), a dipterous insect, looks, as every one knows who has taken any interest in the exploration of Central Africa, not unlike a large ordinary house-fly, has its habitat over a great part of the warmer portions of the continent, especially toward the south and center, and has the power of causing death by its bite of the horse, donkey, cattle, and dog. Cattle sometimes recover, but the others invariably die, after suffering from fever, with emaciation, destruction of the red blood corpuscles, and swelling of the neck, abdomen, or extremities from the presence of coagulable lymph. It was thought that the fatal results were due to a poison formed in the body of the fly, but Surgeon-Major Bruce has recently shown that the tsetse acts merely as a carrier of the poison. Susceptible animals may be bitten with impunity by these flies imported and kept away from the fly country, but die when the flies had previously bitten an affected animal, or when bitten by flies living in the fly country. Bruce found that the blood of animals suffering from "magana," or the fly disease, contained a hematozoon, which is identical with, or at least closely resembles the trypanosoma found in the disease called surra in India. The hematozoon is believed to be the cause of death, but its source is still a mystery.

The tsetse fly may be compared to the mosquito, which has been shown to be a carrier of disease in like manner, and other blood-sucking insects may perhaps be placed in the same category.

The ordinary house-fly is a probable carrier of many infectious diseases, such as fevers; and in countries like Egypt, where it swarms with such determination around diseased eyes that those affected learn scarcely to attempt to brush it away, it is of immense importance as a disseminator of contagious ophthalmia.

Probably more attention given to means for the destruction of insect pests would help to eliminate one of the dangers to the public health, as well as make life more comfortable in warm climates.

